# Generation of sheep with defined *FecB*^*B*^ and *TBXT* mutations and porcine blastocysts with *KCNJ5*^*G151R/*+^ mutation using prime editing

**DOI:** 10.1186/s12864-023-09409-y

**Published:** 2023-06-12

**Authors:** Shiwei Zhou, Laura Johanna Lenk, Yawei Gao, Yuhui Wang, Xiaoe Zhao, Menghao Pan, Shuhong Huang, Kexin Sun, Peter Kalds, Qi Luo, Simon Lillico, Tad Sonstegard, Ute I. Scholl, Baohua Ma, Bjoern Petersen, Yulin Chen, Xiaolong Wang

**Affiliations:** 1 College of Veterinary Medicine, Northwest A&F University, Yangling, 712100 China; 2 Key Laboratory of Animal Genetics, Breeding and Reproduction of Shaanxi Province, College of Animal Science and Technology, Northwest A&F University, Yangling, 712100 China; 3 Institute of Farm Animal Genetics, Friedrich-Loeffler-Institut, 31535 Neustadt, Germany; 4 Department of Animal and Poultry Production, Faculty of Environmental Agricultural Sciences, Arish University, El-Arish, 45511 Egypt; 5 The Roslin Institute and R(D)SVS, University of Edinburgh, Easter Bush Campus, Midlothian, EH25 9RG UK; 6 Recombinetics, St. Paul, MN 55121 USA; 7 Center of Functional Genomics, Berlin Institute of Health at Charité – Universitätsmedizin Berlin, 10115 Berlin, Germany; 8 International Joint Agriculture Research Center for Animal Bio-Breeding, Ministry of Agriculture and Rural Affairs, Yangling, 712100 China

**Keywords:** Prime editing, Genetic improvement, Human disease modeling, Sheep, Pigs

## Abstract

**Background:**

Rewriting the genomes of living organisms has been a long-standing aim in the biological sciences. The revelation of the CRISPR/Cas9 technology has revolutionized the entire biological field. Since its emergence, this technology has been widely applied to induce gene knockouts, insertions, deletions, and base substitutions. However, the classical version of this system was imperfect for inducing or correcting desired mutations. A subsequent development generated more advanced classes, including cytosine and adenine base editors, which can be used to achieve single nucleotide substitutions. Nevertheless, these advanced systems still suffer from several limitations, such as the inability to edit loci without a suitable PAM sequence and to induce base transversions. On the other hand, the recently emerged prime editors (PEs) can achieve all possible single nucleotide substitutions as well as targeted insertions and deletions, which show promising potential to alter and correct the genomes of various organisms. Of note, the application of PE to edit livestock genomes has not been reported yet.

**Results:**

In this study, using PE, we successfully generated sheep with two agriculturally significant mutations, including the fecundity-related *FecB*^*B*^ p.Q249R and the tail length-related *TBXT* p.G112W. Additionally, we applied PE to generate porcine blastocysts with a biomedically relevant point mutation (*KCNJ5* p.G151R) as a porcine model of human primary aldosteronism.

**Conclusions:**

Our study demonstrates the potential of the PE system to edit the genomes of large animals for the induction of economically desired mutations and for modeling human diseases. Although prime-edited sheep and porcine blastocysts could be generated, the editing frequencies are still unsatisfactory, highlighting the need for optimizations in the PE system for efficient generation of large animals with customized traits.

**Supplementary Information:**

The online version contains supplementary material available at 10.1186/s12864-023-09409-y.

## Background

Since the discovery of the CRISPR/Cas system as an adaptive and inheritable immune system in microorganisms, genome-editing tools derived from it have been rapidly developed and widely applied in agricultural and biomedical studies [1, 2]. However, the induction of defined point mutations by the CRISPR/Cas9 system requires double-strand breaks (DSBs) and homology directed repair (HDR) with donor DNA. CRISPR/Cas9, therefore, preferentially produces random nucleotide insertions and deletions at DSB sites, resulting in frameshift mutations [3]. The CRISPR-based cytosine base editors (CBEs) and adenine base editors (ABEs) were developed to achieve base transitions (C → T, G → A, A → G, and T → C) without requiring DSBs [4, 5]. However, the CBE and ABE systems are unable to induce base transversions (C → A, C → G, G → C, G → T, A → C, A → T, T → G, and T → A), insertions, and deletions, and lack precision due to their wide editing windows. In contrast, the emergence of prime editing, a search-and-replace tool, as a new member of the genome-editing toolkit enabled these elusive modifications [6].

Prime editors (PEs) were generated by fusing a reverse transcriptase (RT) to the Cas9 H840A nickase. PEs can induce editing events guided by the prime editing guide RNA (pegRNA), which is derived from a single guide RNA (sgRNA) by tailing its 3’ terminus with primer binding site (PBS) and transcription template sequences [7]. PEs use an RT to directly copy genetic information from an extension on the pegRNA into the target genomic locus, thus enabling all 12 types of base-to-base conversions and small insertions and deletions without requiring DSBs or DNA donors. PEs, initially exemplified by PE1, then PE2 was developed by integrating a more efficient engineered RT, followed by PE3 which was developed by utilizing an additional nicking sgRNA on the opposite DNA strand [6]. PEs have been employed in cells of a wide range of species, including humans, mice, and plants [6, 8, 9]. Notably, PEs have remarkably lower off-target effects than Cas9, fewer byproducts compared to Cas9-initiated HDR, and fewer bystander mutations compared to base editors [6, 10]. To date, PEs have been substantiated in model animals such as mice and zebrafish, achieving editing efficiencies of ~ 10% to 50% [8, 11]. However, the potential of PEs has not yet been verified in large mammals.

The *BMPRIB* gene was first discovered in Booroola Merino sheep and identified as a major gene associated with increased ovulation rates [12]. It affects the differentiation of granulosa cells and promotes follicular development and ovulation. The *FecB*^*B*^ p.Q249R mutation within *BMPR1B* is highly associated with increased ovulation rate and litter size in many sheep breeds [12, 13]. Although we previously induced the p.Q249R mutation in sheep using the ABE system, we observed stubborn bystander mutations within the editing window [14]. *TBXT* is an embryonic nuclear transcription factor regulating mesoderm formation and differentiation [15, 16], that has been highlighted to influence tail development in various organisms. Mutations within *TBXT* were identified to affect the development of the tail vertebrae in dogs, cats, and cattle, leading to a short tail phenotype [17–19]. This phenotype is desired because it obliterates the need for tail docking. In sheep, the *TBXT* c.G334T mutation was highly associated with the short tail phenotype [20–22]. Although we have previously used CRISPR/Cas9-based HDR to induce the *TBXT* p.G112W mutation in Chinese Tan sheep, only indels were observed at the target site (data not shown). Thus, more precise and versatile genome-editing tools, such as PEs, are still required to achieve these economically desired mutations.

As a large mammal, the pig is an attractive model for human diseases, due to its anatomical and physiological similarities to humans. Genome engineered pigs are successfully used in translational biomedical research [23–25]. Primary aldosteronism, the most common cause of secondary hypertension, is characterized by excess production of the adrenal steroid hormone aldosterone. This can be due to aldosterone-producing adenomas (benign adrenal tumors), diffuse hyperplasia, or aldosterone-producing (micro)nodules. Two heterozygous somatic mutations in the *KCNJ5* potassium channel gene (p.G151R and p.L168R) account for about 40% of all aldosterone-producing adenomas [26, 27]. In addition, heterozygous germline mutations in the same gene cause familial hyperaldosteronism type III [26, 28]. The *KCNJ5* gene shows little or no expression in rodent adrenal glands [29], and a mouse model does not seem to reflect the human phenotype [30]. Recently, Vohra et al. showed the suitability of pigs for adrenal research [31]. Precise integration of the p.G151R or p.L168R mutations into porcine fibroblasts, followed by cloning, could speed up the process of generating an animal model for primary aldosteronism.

In this study, we applied for the first time the prime editing system to achieve gene-edited sheep carriers of two economically desired mutations (*FecB*^*B*^ p.Q249R and *TBXT* p.G112W) and gene-edited pig blastocysts carrying a biomedically relevant point mutation (*KCNJ5* p.G151R). This study provides a significant epitome of applying the recently emerged prime editing system to achieve elusive mutations in livestock genomes for agricultural and biomedical applications. This study also provides an applicable reference for the employment of prime editing in farm animals.

## Methods

### Generation of prime-edited sheep

#### Cell culture and transfection

Human embryonic kidney 293 T (HEK293T) cells were cultured in DMEM (Gibco), containing 10% FBS (Gibco) and incubated at 37 °C with 5% CO_2_. When HEK293T cells were cultured to approximately 70% confluence, plasmids were transfected using Lipofectamine® 3000 (Invitrogen) into 6-well plates as previously reported [32]. A total of 375 ng PE2, 125 ng pegRNA, and 40 ng corresponding nicking sgRNA in a form of plasmids were co-transfected into cells. Primers used for genotyping PE3-targeted *FecB*^*B*^ mutation in HEK293T cells are listed in Additional file 2: Table S1. The sequences of pegRNA and sgRNA are listed in Additional file 2: Table S2.

#### Design of pegRNAs and corresponding nicking sgRNAs

**Fig. 1:**
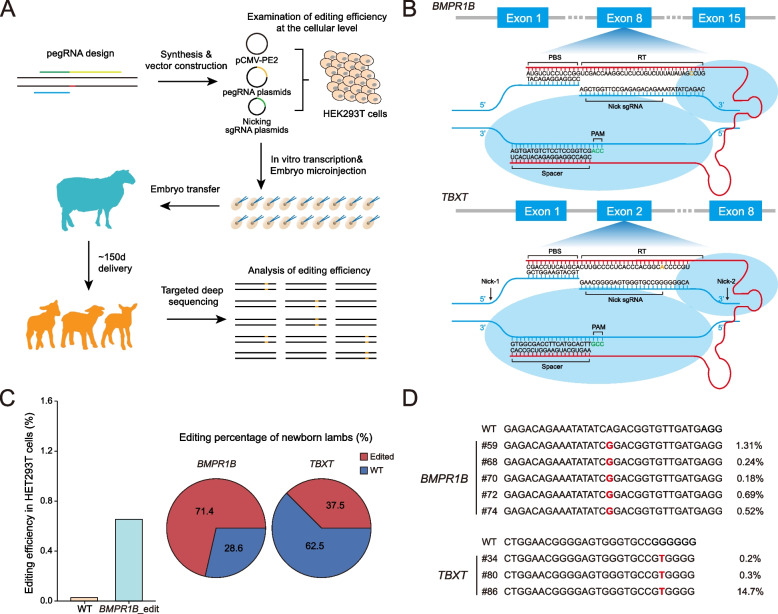
Detection of PE-mediated nucleotide substitutions in sheep. **A** Schematic view of the generation of prime-edited lambs via the PE3 system. pegRNA: prime editing guide RNA and sgRNA: single guide RNA. **B** Schematic view of the target site in the sheep *BMPR1B* and *TBXT* genes. PBS: primer binding site, RT: reverse transcriptase template, and PAM: protospacer adjacent motif. **C** Targeted editing efficiency in HEK293T cells and newborn lambs. WT: wild-type. **D** Sequencing alignments of the DNA fragments with defined mutations in *BMPR1B*-edited and *TBXT*-edited animals

The pegRNAs targeting the sheep *FecB*^*B*^ mutation (p.Q249R) and *TBXT* mutation (p.G112W) were designed (Fig. 1B) according to the recommendations of Anzalone et al. [6]. The sequences of pegRNAs and sgRNAs used for in vitro transcription are listed in Additional file 2: Table S3.

#### In vitro transcription of the PE system

The PE2 plasmid was linearized by the *Pme*I enzyme (NEB). The linearized plasmid was used for in vitro transcription with the mMESSAGE mMACHINE T7 Ultra Kit (Ambion). The PE2 mRNA was purified by the Mini Kit (Qiagen) according to the manufacturer’s instructions. All in vitro transcription templates of pegRNAs and sgRNAs were generated using the PCR amplification of the U6-promoted sgRNA-expressing PGL3-U6 vector (Addgene No. #51,133; http://n2t.net/addgene:51133) [33]. MEGAshortscript T7 transcription kit (Ambion) was used for the in vitro transcription of pegRNAs and sgRNAs. The pegRNAs and sgRNAs were purified by the MEGAclear Kit (Ambion) according to the manufacturer’s instructions. Primers used for in vitro transcription of PE2, pegRNAs, and sgRNAs are listed in Additional file 2: Table S4.

#### Generation of PE-induced gene-edited sheep

Healthy ewes with normal estrous cycles were selected as donors for zygote collection. The superovulation treatment of donors was performed as previously described [14]. Briefly, an EAZI-BREED controlled internal drug release (CIDR) Sheep and Goat Device was inserted into the vagina of the donor ewes for 12 days. The CIDR Device contained 300 mg of progesterone. The superovulation was carried out 60 h prior to the removal of the CIDR Device. Each donor ewe was naturally mated three times (the first mating at 12 h after the initial estrus, and then subsequent matings were carried out at 12 h intervals). Zygotes at the one-cell stage were collected by a surgical operation and immediately transferred to Quinn’s Advantage Cleavage medium (Sage Biopharma, Toronto, Canada). The mixture of PE2 mRNA (100 ng/μL), pegRNA (40 ng/μL), and sgRNAs (10 ng/μL) was co-injected into the cytoplasm of the collected zygotes using the Eppendorf FemtoJet system. The injection pressure, compensatory pressure, and time parameter were 45 kPa, 7 kPa, and 0.1 s, respectively. Injected embryos were cultured for 6 ~ 8 h and subsequently transferred into surrogates as previously described [34]. Pregnancy was confirmed by observing the estrous behavior of the surrogates at every ovulation cycle. After ~ 150 days of pregnancy, newborn lambs were obtained and sufficient care was given.

#### Genotyping of generated founders

Peripheral venous blood samples from two-week-old lambs were collected to extract genomic DNA. PCR amplification was used to prepare genomic DNA samples for targeted deep sequencing. The primers used for genotyping are listed in Additional file 2: Table S5.

#### Prediction of off-target sites

Potential off-target sites within three mismatches and NGG protospacer adjacent motif (PAM) sites were predicted for pegRNAs and nicking sgRNAs using the online available software Cas-OFFinder [35] (Additional file 2: Tables S6 and S7). For *TBXT* and *BMPR1B* genes, 16 and 14 potential off-target sites were predicted, respectively. The primers for amplifying the off-target sites for Sanger and targeted deep sequencing are listed in Additional file 2: Tables S8 and S9.

#### Captured deep sequencing

Target mutations and potential off-target sites were amplified using a KAPA HiFi HotStart PCR Kit (#KK2501; KAPA Biosystems, Wilmington, MA, USA) to generate deep sequencing libraries as previously described [36]. The pool of PCR amplicons was sequenced using MiniSeq with the TruSeq HT Dual Index system (Illumina, San Diego, CA, USA).

### Generation of prime-edited porcine blastocysts

#### pegRNA and sgRNA design, and plasmid cloning

pegRNA and sgRNA for the mutation of the porcine *KCNJ5* gene were designed using the web design tool pegFinder (http://pegfinder.sidichenlab.org/) [37], using the recommended parameters. The pCMV-PE2 was a gift from David Liu (Addgene No. #132,775; http://n2t.net/addgene:132775) [6]. PegRNA sequences (Additional file 2: Table S10) were integrated into the pegRNA expression plasmid pU6-pegRNA-GG-acceptor, a gift from David Liu (Addgene No. #132,777; http://n2t.net/addgene:132777) [6] and sgRNA sequences for the PE3b approach were integrated into the BPK1520 plasmid, a gift from Keith Joung (Addgene No. #65,777; http://n2t.net/addgene:65777) [38], in accordance with the cloning protocols supplied by Anzalone et al. [6] and the Joung Lab gRNA Cloning Protocol (Version 1.2 – October 2015), respectively.

#### Transfection of porcine kidney fibroblasts

Porcine kidney fibroblasts were cultured in Dulbecco’s modified Eagle’s medium with 1% penicillin/streptomycin, nonessential amino acids, sodium pyruvate, and 10% fetal calf serum. Approximately 3 × 10^6^ cells were transfected with a total of 10 μg of the PE2 expression plasmid pCMV-PE2, the pegRNA expression vector, and the sgRNA expression vector in a 9:3:1 ratio. For transfection via electroporation, the Neon Transfection System (Invitrogen, ThermoFisher Scientific) was used (100 μL Kit) under the following conditions: 1650 V, 3 Pulse, 10 ms, and cells were cultured in 30% fetal calf serum afterwards.

#### Digital PCR (dPCR)

For the dPCR analysis, a TaqMan® Custom SNP Genotyping Assays, containing two primers (Ex2_p_KCNJ5_F: CTGCCTTCTTGTTCTCCATCGA, Ex2_p_KCNJ5_R: TCTGGACACTTCTCGGTGATCA) and two probes (VIC-labeled Ex2_p_KCNJ5_V: ACGACCATCGGGTACG and FAM-labeled Ex2_p_KCNJ5_M: ACGACCATCAGGTACG) were used. The reaction volume of 14.5 μL, containing 7.25 μL QuantStudio3D Digital PCR Master Mix v2 (ThermoFisher Scientific), 0.725 μL SNP assay, 1.525 μL nuclease-free water, and 5 μL diluted genomic DNA was loaded onto the chip using the QuantStudio™ 3D Digital PCR Chip Loader, and chips were thermocycled under the standard thermocycling conditions recommended by the supplier (Pro Flex 2 × Flat PCR-System PCR Method), but with 45 cycles. Afterwards, the chips were analyzed by the QuantStudio 3D Digital PCR Instrument and the rare mutation analysis.

#### PCR, restriction enzymatic digestion, and Sanger sequencing

For PCR, DNA was isolated from cells using a non-commercial cell lysis buffer, containing 0.02% SDS (Roth), 0.05 mg/mL Proteinase K (Thermo Fisher Scientific), and 20 mM Tris HCl (Roth). PCR for restriction enzymatic digestion and Sanger sequencing was performed using *KCNJ5* specific primers (Additional file 2: Table S11) for analysis of cells. Restriction enzymatic digestion was performed using *Dde*I (NEB) for 60 min at 37 °C. The analysis of blastocysts using nested PCR was conducted using the following primers: *KCNJ5*-FW2 and *KCNJ5*-RV2 (Additional file 2: Table S11).

#### Somatic nuclear cell transfer (SCNT)

SCNT was performed as previously described [39]. Blastocysts were collected at day 6 or 7 after SCNT, and DNA was isolated by incubating single blastocysts in 15 μL cell lysis buffer at 55 °C for 1 h in a thermomixer, followed by 95 °C for 12 min.

#### pGEM-T easy

For allele specific sequencing, PCR products of blastocysts were subcloned into the pGEM®-T easy vector system (Promega) according to the manufacturer’s protocol. After ligation, the plasmids were transformed into NEB® 5-alpha competent *E. coli* and single colonies were picked, and PCR for Sanger sequencing was performed.

## Results and discussion

### Examination of prime editing efficiency in HEK293T cells

To generate gene-edited sheep with the *FecB*^*B*^ and *TBXT* mutations using PEs, we first validated the editing versatility of PEs at the *FecB*^*B*^ locus in HEK293T cells (Fig. 1A). The HEK293T cell line was selected for in vitro validation based on its high transfection efficiency compared to sheep fibroblasts. PE3 was selected to verify the gene target efficiency, since it outperforms PE2 on editing efficiency [6, 8]. The pCMV-PE2 plasmid and plasmids encoding pegRNA and nicking sgRNA were co-transfected into HEK293T cells. Genomic DNA was extracted from transfected cells and subjected to PCR amplification for targeted deep sequencing (Additional file 2: Table S1). The editing efficiency at the target site was merely 0.67% (Fig. 1C). This frequency is much lower than those editing frequencies observed in other recent studies [6, 8, 40, 41]. This may be due to the absence of an enrichment step of transfected cells and the variety of prime editing efficiency at different genomic sites. However, we proceeded further to examine the PE efficiency at the embryonic and animal levels to provide more insights regarding the PE efficiency in vivo.

### Generation of prime-edited sheep with the *FecB*^*B*^ and *TBXT* mutations

To produce gene-edited lambs with PE3, PE2 mRNA and different pegRNAs were co-injected with corresponding nicking sgRNAs into the cytoplasm of one-cell stage zygotes using an Eppendorf FemtoJet system (Fig. 1B), as we previously conducted [14]. Twenty-five mated Chinese Tan sheep donors were superovulated and produced 268 one-cell stage fertilized oocytes. Of the 268 microinjected embryos, 262 (122 for *FecB*^*B*^ and 140 for *TBXT*) were cultured in an ideal developmental condition and were transplanted into 45 recipients. After a full-term gestation period (~ 150 days), 15 lambs [seven (#51, #59, #68, #70, #72, #74, and #78) with targeted *FecB*^*B*^ and eight (#34, #43, #54, #56, #63, #80, #84, and #86) with targeted *TBXT*] were born (Table 1).

**Table 1:** Summary of the lambs obtained with targeted point mutations via the PE3 system

Item	Number	
	**Targeted *BMPR1B***	**Targeted *TBXT***
Donors	25	
Collected one-cell stage zygotes	268	
PE-pegRNA-sgRNA injected embryos	268	
Transferred embryos	262	
Recipient ewes	45	
Pregnant recipients	15	
Newborns	7	8
Expected defined substitution	5	3
Wild-types	2	5

Genomic DNA was extracted from the 15 lambs and PCR was performed for targeted deep sequencing. The results revealed that five lambs (#59, #68, #70, #72, and #74) out of seven (71.4%) were edited at the target site (p.Q249R) in the *BMPR1B* gene (Fig. 1C); however, the edited lambs showed varied mutation frequencies (i.e., the percentage of edited alleles) ranging from 0.18 to 1.31% (Fig. 1D and Additional file 1: Fig. S1). For the target site in *TBXT* (p.G112W), three lambs (#34, #80, and #86) out of eight (37.5%) were edited (Fig. 1C), with mutation frequencies ranging from 0.2 to 14.7% (Fig. 1D and Additional file 1: Fig. S1). To our knowledge, the obtained editing frequencies in mutant sheep are relatively equivalent to those reported in some prime editing studies in plants, mice, and *Drosophila* [8, 9, 40]. Although the mutation frequency is low, the success of PE3 to access and edit the sheep genome supports the potential of more developed PE systems for the generation of large animals with defined point mutations. We anticipate that the future optimizations of PEs will promote its applications in large animals for editing currently inaccessible agriculturally significant variants.

### Evaluation of off-target activities in prime-edited sheep

To evaluate the off-target effects in PE-edited animals, 14 and 16 putative off-target sites with NGG PAM for pegRNAs and nicking sgRNAs were predicted for *FecB*^*B*^ and *TBXT* target sites, respectively (Additional file 2: Tables S6 and S7). Using the genomic DNA from the five *FecB*^*B*^-edited and three *TBXT*-edited founders, we amplified the regions around the putative off-target sites. Sanger sequencing of the eight prime-edited lambs revealed undetectable off-target mutations at all the examined sites (Additional file 1: Fig. S2-S5). Similarly, targeted deep sequencing revealed no potential off-target mutations in the edited lambs (Additional file 3: Tables S1 and S2). Compared to base editing and Cas9 techniques [42–44], PEs produce almost no off-target events [8, 40] with the RT template and PBS sequences serving as a dual checkpoint to prevent unintended editing. Taken together, these results demonstrate the precision and specificity of PEs for precise genome editing in vivo.

### Examination of prime editing efficiency in porcine kidney fibroblasts

**Fig. 2:**
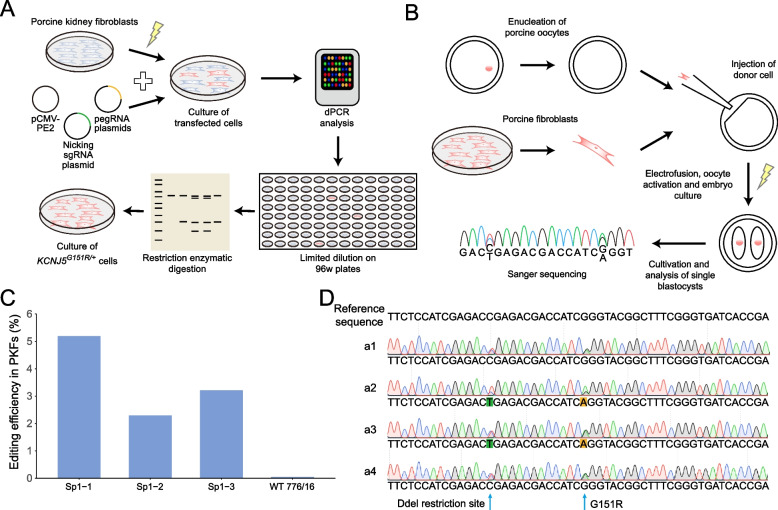
Detection of the successfully integrated *KCNJ5* p.G151R mutation in porcine fibroblasts and generation of prime-edited blastocysts. **A** Schematic view of the generation of *KCNJ5*^*G151R/*+^ cell clones. **B** Schematic view of the generation of prime-edited porcine blastocysts. **C** Editing efficiency determined by dPCR in three independent transfections. WT 776/16: unedited wild-type control. **D** Sanger sequence alignment of four blastocysts with heterozygous integration of both desired mutations (sequences of all blastocysts are shown in Additional file 1: Fig. S7)

To expand the application of the PE system, we applied it to integrate point mutations into the porcine genome. A pegRNA targeting the porcine *KCNJ5* gene was designed, aiming to introduce the p.G151R mutation and an additional silent mutation for the selection of successfully edited cells by PCR and restriction enzymatic digestion with *Dde*I, as well as a sgRNA for the PE3b approach (Fig. 2A and Additional file 2: Table S10). The pCMV-PE2 plasmid and plasmids encoding pegRNA and nicking sgRNA were co-transfected into porcine kidney fibroblasts. To determine the editing efficiency, we isolated DNA from the transfected cells and performed dPCR. Two probes for a SNP assay were designed to detect *KCNJ5* p.G151R and wild-type copies. Three replications under the same transfection conditions resulted in 5.2%, 2.3%, and 3.2% edited *KCNJ5* copies, respectively (Fig. 2C and Additional file 1: Fig. S6). To confirm the correct integration of both mutations, we performed limited dilution of cells produced by the approach with the highest editing efficacy and identified a cell clone carrying the heterozygous *KCNJ5*^*G151R/*+^ and the silent *Dde*I mutation, as intended. These results confirm the ability of the PE system to access and edit the porcine genome for the induction of intended point mutations.

### Generation of prime-edited porcine blastocysts with the *KCNJ5* p.G151R mutation

The cell clone carrying the heterozygous *KCNJ5*^*G151R/*+^ and the silent *Dde*I mutation was used as a cell donor for SCNT (Fig. 2B). A total of 173 cloned complexes were generated in three SCNT sessions. Ninety-eight complexes were cultured after successful injection of cells into the perivitelline space of enucleated oocytes and fusion and activation of the complexes. Of these 98 reconstructed complexes, 10 developed to blastocysts after 6 to 7 days of in vitro culture (blastocyst rate of 10%, Additional file 2: Table S12). The lysate of the isolated blastocysts was used for PCR and nested PCR. Subsequently, the PCR product was genotyped with Sanger sequencing, and all blastocysts showed the expected genotype (Fig. 2D and Additional file 1: Fig. S7). Allele-specific sequencing validated the successful editing of only one allele, resulting in a heterozygous genotype (Additional file 1: Fig. S8A and S8B). Collectively, these results show that the editing of the *KCNJ5* locus in pig fibroblasts had a higher efficiency compared to the *FecB*^*B*^ locus in HEK293T cells. We also note that a high variability between different target sites was shown as previously highlighted [9]. Taken together, these results underline the prospect of the PE system and its future advanced classes to play an important role in the generation of genome-engineered pigs.

## Conclusions

In summary, we report the first PE application in livestock. We precisely generated sheep with two single base conversions at two different loci and pig blastocysts with two single base conversions at a single targeted gene. The efficiency of defined point mutations was up to 71.4% (5/7) at the animal level and the editing frequency was up to 14.7%. For porcine fibroblasts, the editing efficiency was up to 5.2%, yet not matching the efficiencies described in other species. Although the PE system showed high precision, further improvements are highly required to enhance the editing frequency. Collectively, our results validated the precision and potential of the PE system for multiplexed genome editing for animal breeding and translational research, and the functional validation of key SNPs in livestock.

## Supplementary Information

**Additional file 1:** **Fig S1. **Editing efficiency in the generated lambs as shown by targeted deep sequencing.** Fig. S2. **Detection of potential off-target sites by Sanger sequencing in founder animals. Five potential off-target sites (Spacer OT1-OT5) were predicted by Cas-OFFinder in *FecB*^*B*^-edited lambs. Sanger sequencing was used to determine variations at predicted target sites for the five founder animals. **Fig. S3. **Detection of potential off-targeted sites by Sanger sequencing in founder animals. Nine potential off-target sites (Nick OT1-OT9) were predicted by Cas-OFFinder in *FecB*^*B*^-edited lambs. Sanger sequencing was used to determine variations at predicted target sites for the five founder animals. **Fig. S4. **Detection of potential off-targeted sites by Sanger sequencing in founder animals. A potential off-target site (Spacer OT1) was predicted by Cas-OFFinder in *TBXT*-edited lambs. Sanger sequencing was used to determine variations at predicted target sites for the three founder animals. **Fig. S5.** Detection of potential off-targeted sites by Sanger sequencing in founder animals. Fifteen potential off-target sites (Nick1 OT1-OT8 and Nick2 OT1-OT7) were predicted by Cas-OFFinder in *TBXT*-edited lambs. Sanger sequencing was used to determine variations at predicted target sites for the three founder animals. **Fig. S6.** Digital PCR of PE edited cell populations (Sp1-1, Sp1-2, and Sp1-3) and an unedited wild type control. (a, d, g, j, and m) chip view by calls. (b, c, e, f, h, I, k, l, n, and o) yellow: no amplification, red: VIC reporter dye signal (KCNJ5 WT probe), blue: FAM reporter dye signal (KCNJ5 G151R probe), green: FAM + VIC reporter dye signals. (b) Two-dimensional scatter plot of digital PCR SNP assay and (c) histogram from unedited wild type control cells. (e) Two-dimensional scatter plot of digital PCR SNP assay and (f) histogram from replicate Sp1-1. (h) Two-dimensional scatter plot of digital PCR SNP assay and (i) histogram from replicate Sp1-1 (repetition of dPCR chip). (k) Two-dimensional scatter plot of digital PCR SNP assay and (l) histogram from replicate Sp1-2. (n) Two-dimensional scatter plot of digital PCR SNP assay and (o) histogram from replicate Sp1-3. **Fig. S7. **Sanger sequencing of all ten porcine blastocysts shows a heterozygous p.G151R mutation next to the silent mutation of the *Dde*I restriction enzymatic digestion site. **Fig. S8. **Sanger sequencing of colonies after cloning of blastocyst PCR products into the pGEM-T Easy vector. (a) WT1b_3A_a3 and (b) WT1b_3A_a5 show overlapping sequences besides sequences containing both desired mutations and sequences matching to the wild-type reference sequence.

**Additional file 2:** **Table S1.** List of primers used for genotyping and amplifying PE3-targeted FecB^B^ fragment in HEK293T cells. **Table S2.** Sequences of pegRNA and sgRNA used in human HEK293T cells. **Table S3.** Sequences of pegRNAs and sgRNAs used in sheep. **Table S4.** List of primers used for in vitro transcription. **Table S5.** List of primers for genotyping and amplifying PE3-targeted *BMPR1B* and *TBXT* fragments in newborn lambs. **Table S6.** List of predicted off-target sites for PE3-targeted *BMPR1B*. **Table S7.** List of predicted off-target sites for PE3-targeted *TBXT*. **Table S8.** List of primers for genotyping and amplifying predicted off-target site fragments in FecB^B^-edited sheep. **Table S9.** List of primers for genotyping and amplifying predicted off-target site fragments in *TBXT*-edited sheep. **Table S10.** Sequences of pegRNAs and sgRNAs used in pigs. **Table S11.** List of primers for genotyping and amplifying PE3-targeted *KCNJ5* in porcine kidney fibroblasts and blastocysts. **Table S12.** Number of blastocysts generated per SCNT session using *KCNJ5*^G151R/+^ porcine kidney fibroblasts as donor cells.

**Additional file 3:** **Table S1.** The targeted deep sequencing-based analysis of the off-target site in *BMPR1B*-edited lambs. **Table S2.** The targeted deep sequencing-based analysis of the off-target sites in *TBXT*-edited lambs.

## Data Availability

All relevant results are within this article and its additional files. The raw targeted deep sequencing data is available at the NCBI SRA database under the BioProject ID: PRJNA562971.

## Abbreviations

PEs: Prime editors
DSBs: Double-strand breaks
HDR: Homology directed repair
CBEs: Cytosine base editors
ABEs: Adenine base editors
RT: Reverse transcriptase
pegRNA: Prime editing guide RNA
sgRNA: Single guide RNA
PBS: Primer binding site
CIDR: EAZI-BREED controlled internal drug release
PAM: Protospacer adjacent motif
dPCR: Digital PCR
SCNT: Somatic nuclear cell transfer
WT: Wild-type
