# Supplementary material for: Generation of sheep with defined FecBB and TBXT mutations and porcine blastocysts with KCNJ5G151R/+ mutation using prime editing
Source: BMC Genomics. 2023 Jun 12;24:313. doi: 10.1186/s12864-023-09409-y (PMC10258939; doi:10.1186/s12864-023-09409-y)
Supplement: Supplementary file 1 — Additional file 1: Fig S1. Editing efficiency in the generated lambs as shown by targeted deep sequencing. Fig. S2. Detection of potential off-target sites by Sanger sequencing in founder animals. Five potential off-target sites (Spacer OT1-OT5) were predicted by Cas-OFFinder in FecBB-edited lambs. Sanger sequencing was used to determine variations at predicted target sites for the five founder animals. Fig. S3. Detection of potential off-targeted sites by Sanger sequencing in founder animals. Nine potential off-target sites (Nick OT1-OT9) were predicted by Cas-OFFinder in FecBB-edited lambs. Sanger sequencing was used to determine variations at predicted target sites for the five founder animals. Fig. S4. Detection of potential off-targeted sites by Sanger sequencing in founder animals. A potential off-target site (Spacer OT1) was predicted by Cas-OFFinder in TBXT-edited lambs. Sanger sequencing was used to determine variations at predicted target sites for the three founder animals. Fig. S5. Detection of potential off-targeted sites by Sanger sequencing in founder animals. Fifteen potential off-target sites (Nick1 OT1-OT8 and Nick2 OT1-OT7) were predicted by Cas-OFFinder in TBXT-edited lambs. Sanger sequencing was used to determine variations at predicted target sites for the three founder animals. Fig. S6. Digital PCR of PE edited cell populations (Sp1-1, Sp1-2, and Sp1-3) and an unedited wild type control. (a, d, g, j, and m) chip view by calls. (b, c, e, f, h, I, k, l, n, and o) yellow: no amplification, red: VIC reporter dye signal (KCNJ5 WT probe), blue: FAM reporter dye signal (KCNJ5 G151R probe), green: FAM + VIC reporter dye signals. (b) Two-dimensional scatter plot of digital PCR SNP assay and (c) histogram from unedited wild type control cells. (e) Two-dimensional scatter plot of digital PCR SNP assay and (f) histogram from replicate Sp1-1. (h) Two-dimensional scatter plot of digital PCR SNP assay and (i) histogram from replicate Sp1-1 (repetition of dPC [file 12864_2023_9409_MOESM1_ESM.docx]

**Additional file 1**

**Supplementary Figures**

**
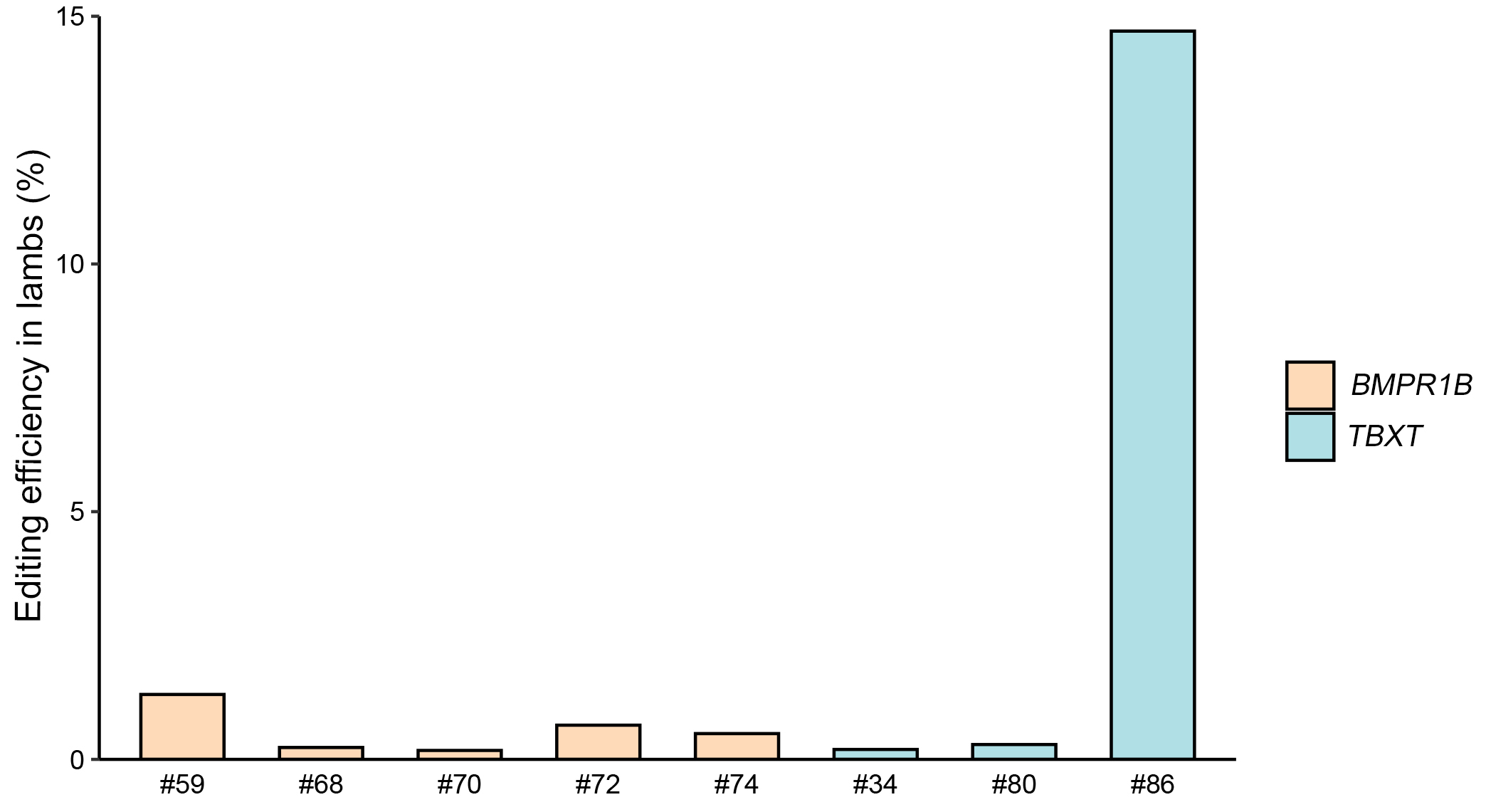
**

**Fig S1.** Editing efficiency in the generated lambs as shown by targeted deep sequencing.


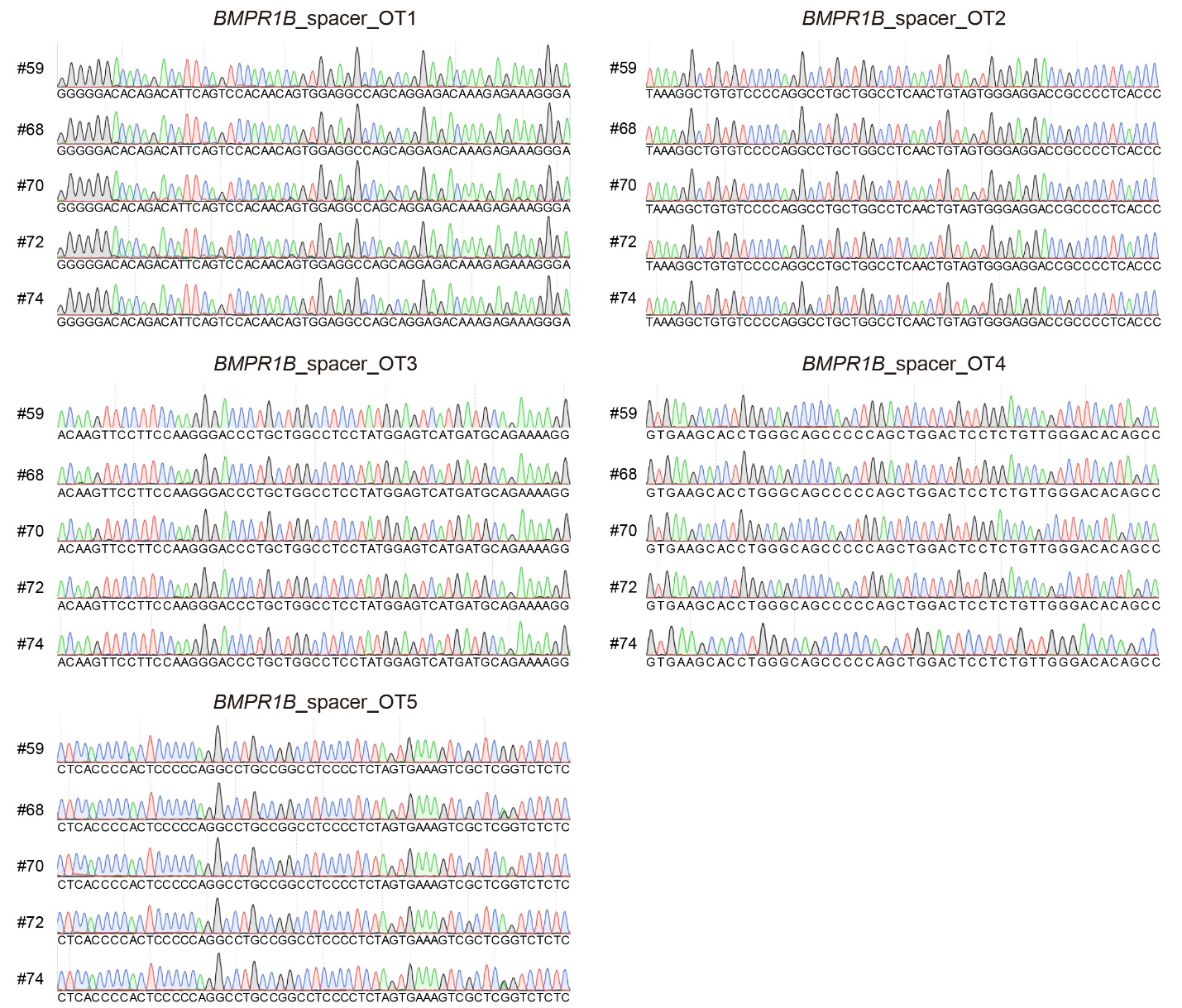


**Fig. S2.** Detection of potential off-target sites by Sanger sequencing in founder animals. Five potential off-target sites (Spacer OT1-OT5) were predicted by Cas-OFFinder in *FecB^B^*-edited lambs. Sanger sequencing was used to determine variations at predicted target sites for the five founder animals.

**
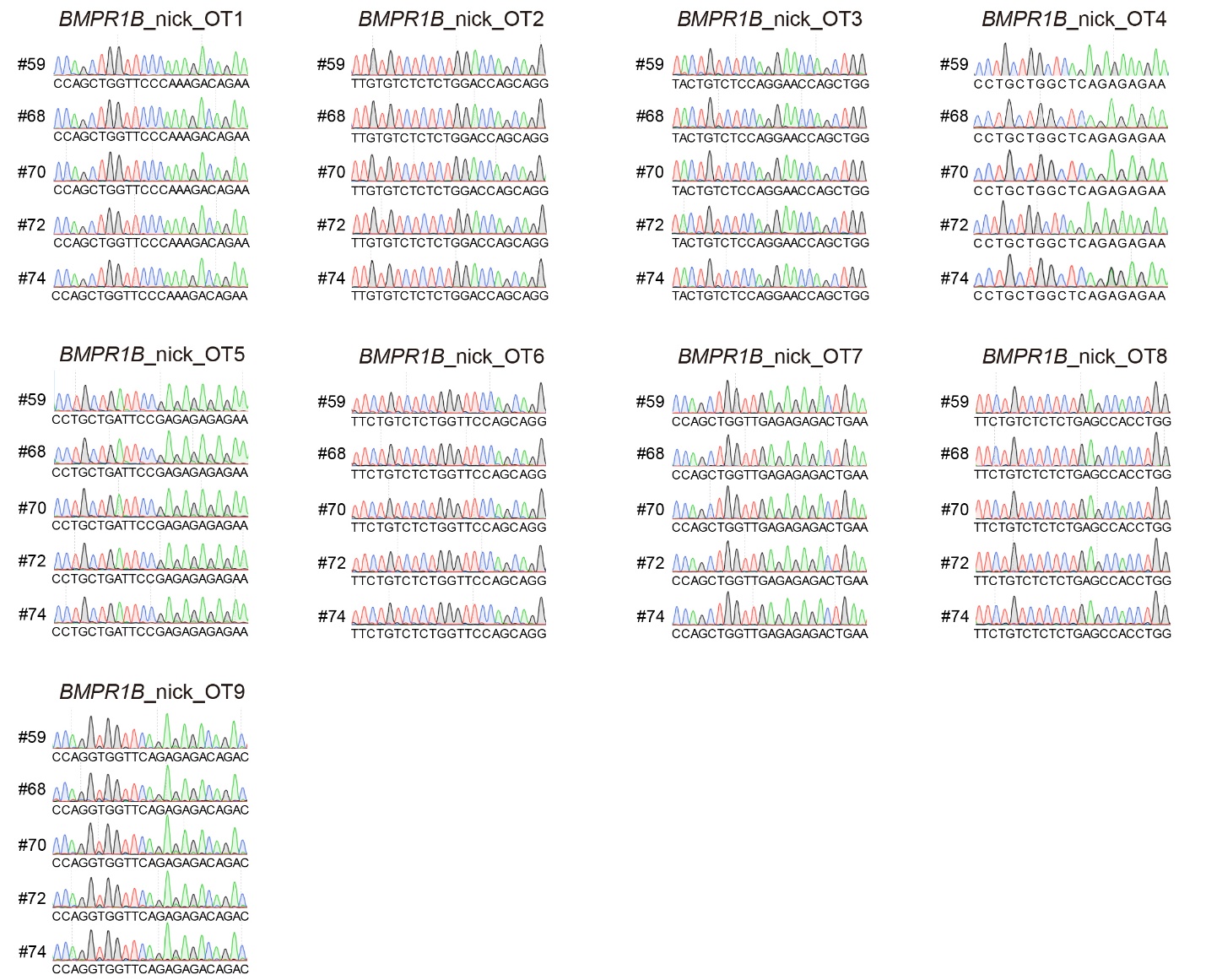
**

**Fig. S3.** Detection of potential off-targeted sites by Sanger sequencing in founder animals. Nine potential off-target sites (Nick OT1-OT9) were predicted by Cas-OFFinder in *FecB^B^*-edited lambs. Sanger sequencing was used to determine variations at predicted target sites for the five founder animals.

**
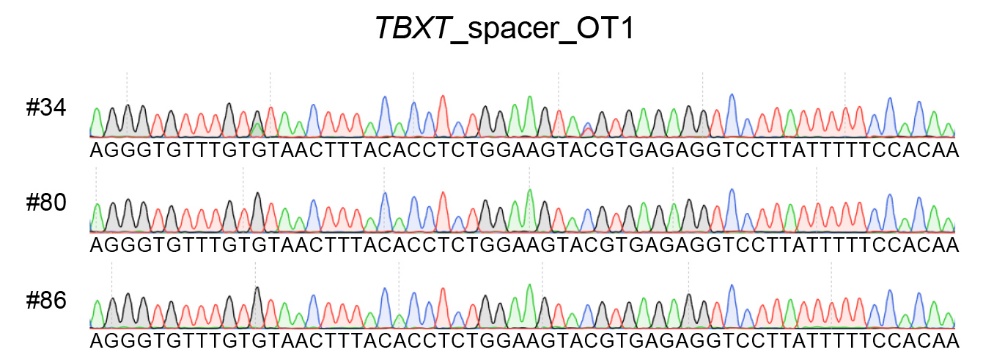
**

**Fig. S4.** Detection of potential off-targeted sites by Sanger sequencing in founder animals. A potential off-target site (Spacer OT1) was predicted by Cas-OFFinder in *TBXT*-edited lambs. Sanger sequencing was used to determine variations at predicted target sites for the three founder animals.

**
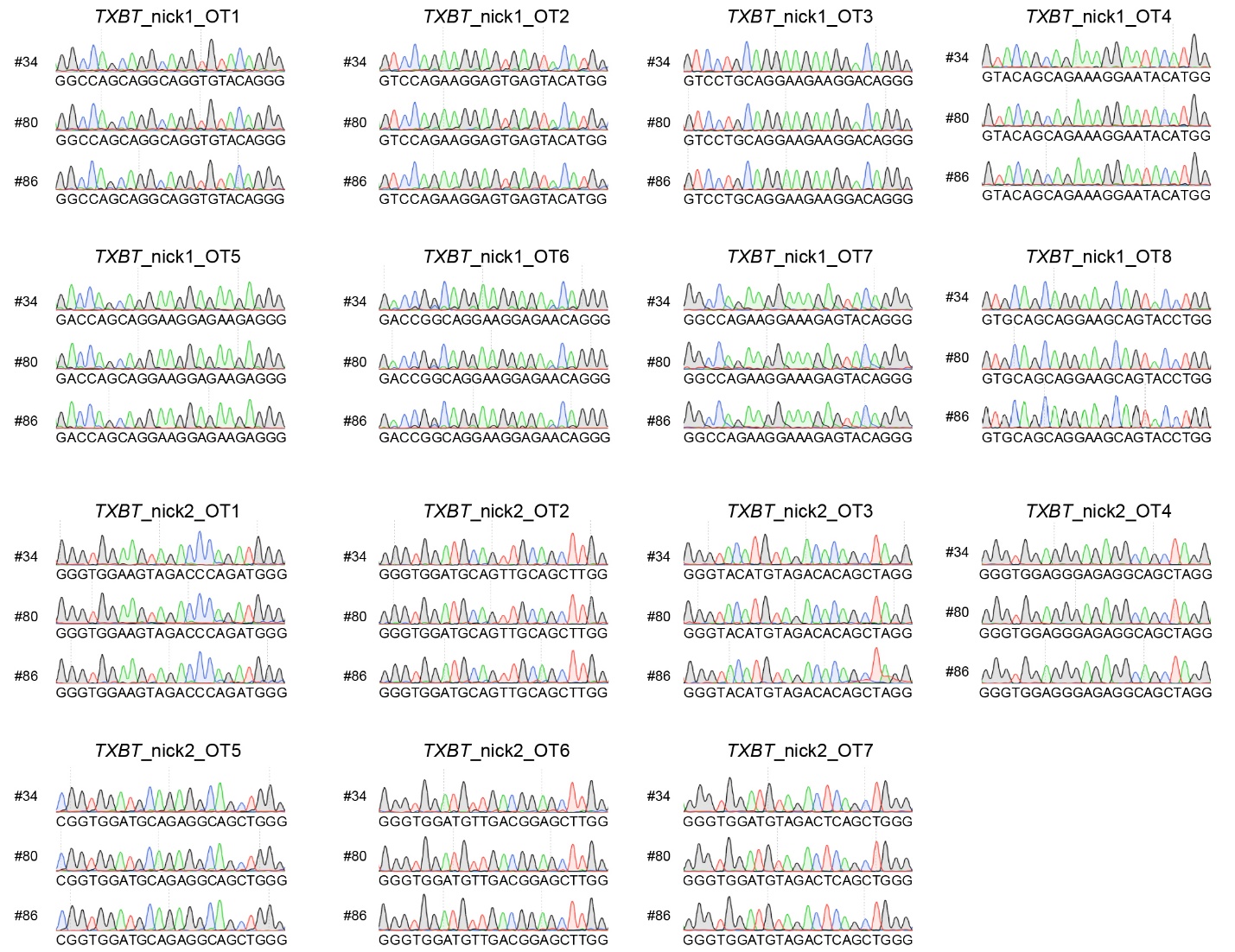
**

**Fig. S5.** Detection of potential off-targeted sites by Sanger sequencing in founder animals. Fifteen potential off-target sites (Nick1 OT1-OT8 and Nick2 OT1-OT7) were predicted by Cas-OFFinder in *TBXT*-edited lambs. Sanger sequencing was used to determine variations at predicted target sites for the three founder animals.


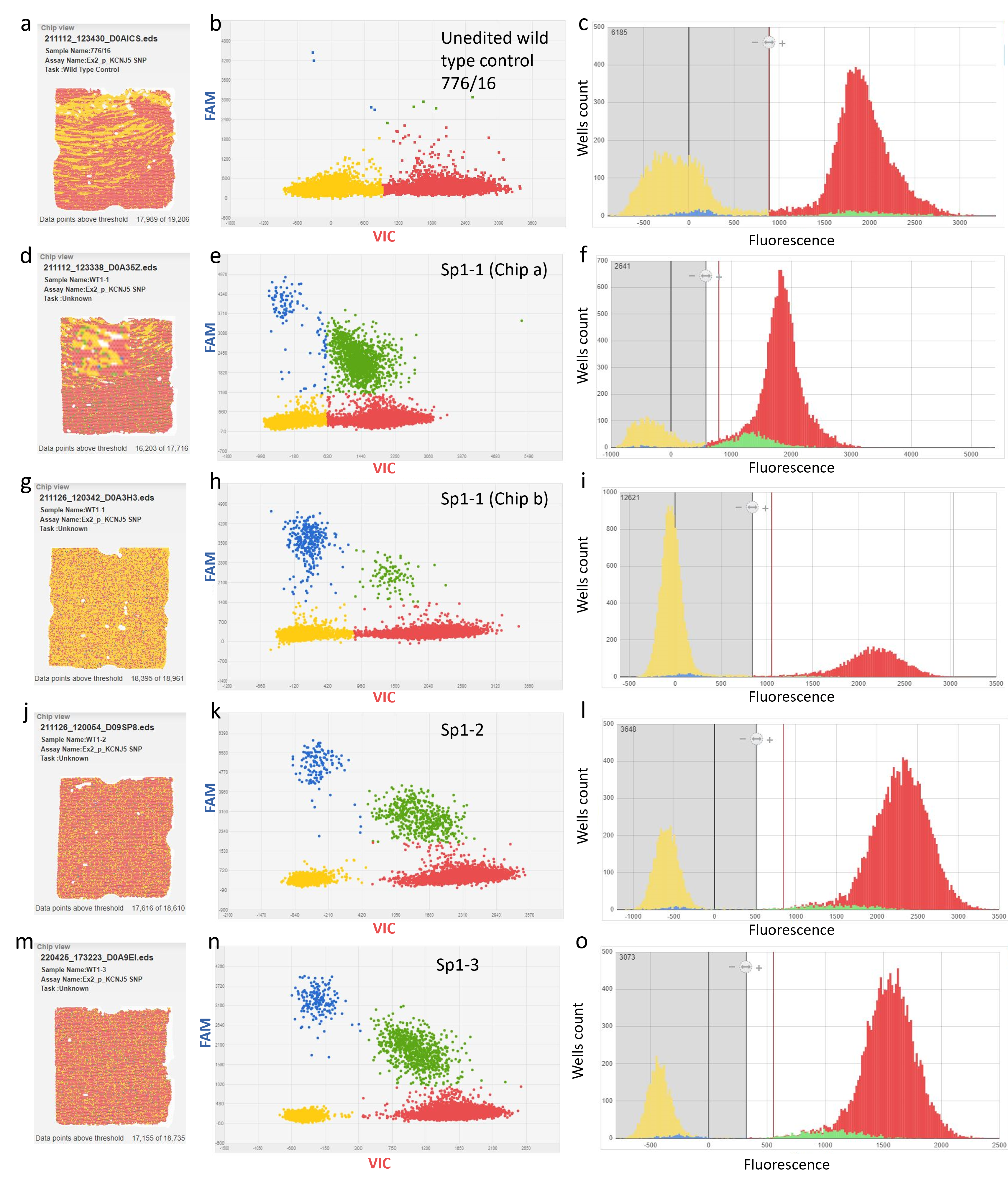


**Fig. S6.** Digital PCR of PE edited cell populations (Sp1-1, Sp1-2, and Sp1-3) and an unedited wild type control. (a, d, g, j, and m) chip view by calls. (b, c, e, f, h, I, k, l, n, and o) yellow: no amplification, red: VIC reporter dye signal (KCNJ5 WT probe), blue: FAM reporter dye signal (KCNJ5 G151R probe), green: FAM + VIC reporter dye signals. (b) Two-dimensional scatter plot of digital PCR SNP assay and (c) histogram from unedited wild type control cells. (e) Two-dimensional scatter plot of digital PCR SNP assay and (f) histogram from replicate Sp1-1. (h) Two-dimensional scatter plot of digital PCR SNP assay and (i) histogram from replicate Sp1-1 (repetition of dPCR chip). (k) Two-dimensional scatter plot of digital PCR SNP assay and (l) histogram from replicate Sp1-2. (n) Two-dimensional scatter plot of digital PCR SNP assay and (o) histogram from replicate Sp1-3.


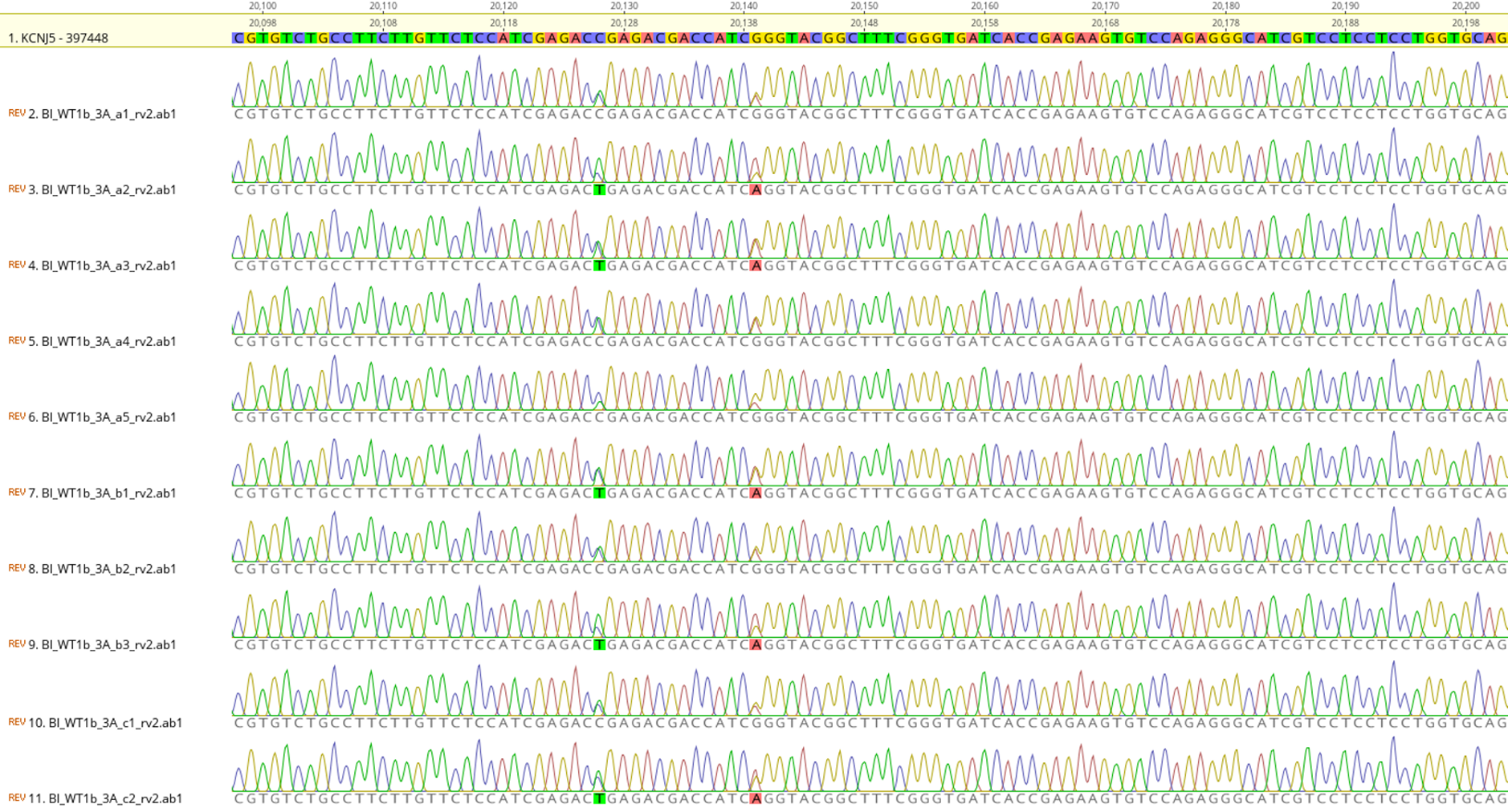
 **Fig. S7.** Sanger sequencing of all ten porcine blastocysts shows a heterozygous p.G151R mutation next to the silent mutation of the *Dde*I restriction enzymatic digestion site.


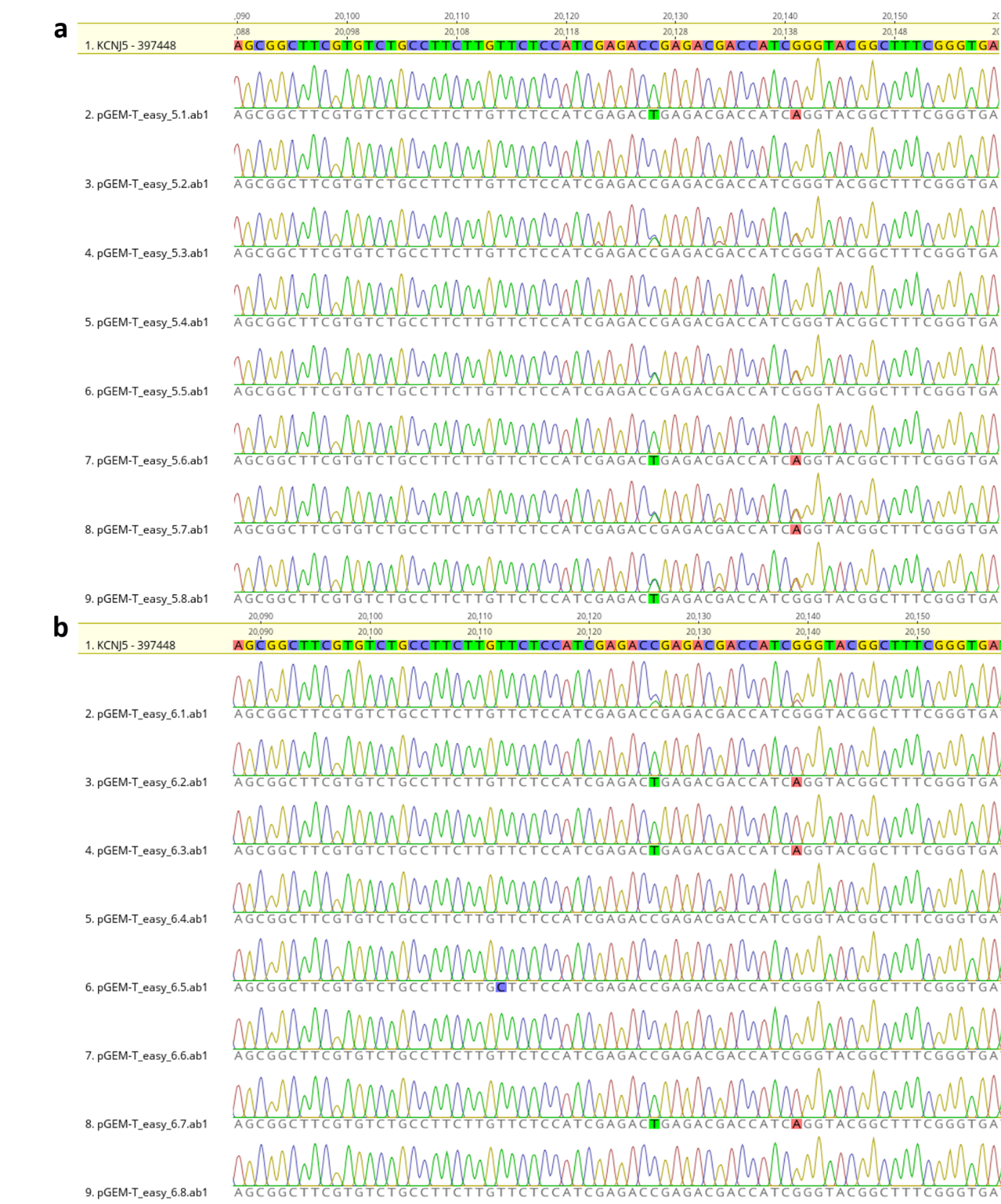


**Fig. S8.** Sanger sequencing of colonies after cloning of blastocyst PCR products into the pGEM-T Easy vector. (a) WT1b_3A_a3 and (b) WT1b_3A_a5 show overlapping sequences besides sequences containing both desired mutations and sequences matching to the wild-type reference sequence.
