# Supplementary material for: Generation of sheep with defined FecBB and TBXT mutations and porcine blastocysts with KCNJ5G151R/+ mutation using prime editing
Source: BMC Genomics. 2023 Jun 12;24:313. doi: 10.1186/s12864-023-09409-y (PMC10258939; doi:10.1186/s12864-023-09409-y)
Supplement: Supplementary file 2 — Additional file 2: Table S1. List of primers used for genotyping and amplifying PE3-targeted FecBB fragment in HEK293T cells. Table S2. Sequences of pegRNA and sgRNA used in human HEK293T cells. Table S3. Sequences of pegRNAs and sgRNAs used in sheep. Table S4. List of primers used for in vitro transcription. Table S5. List of primers for genotyping and amplifying PE3-targeted BMPR1B and TBXT fragments in newborn lambs. Table S6. List of predicted off-target sites for PE3-targeted BMPR1B. Table S7. List of predicted off-target sites for PE3-targeted TBXT. Table S8. List of primers for genotyping and amplifying predicted off-target site fragments in FecBB-edited sheep. Table S9. List of primers for genotyping and amplifying predicted off-target site fragments in TBXT-edited sheep. Table S10. Sequences of pegRNAs and sgRNAs used in pigs. Table S11. List of primers for genotyping and amplifying PE3-targeted KCNJ5 in porcine kidney fibroblasts and blastocysts. Table S12. Number of blastocysts generated per SCNT session using KCNJ5G151R/+ porcine kidney fibroblasts as donor cells. [file 12864_2023_9409_MOESM2_ESM.docx]

**Additional file 2**

**Supplementary Tables**

**Table S1.** List of primers used for genotyping and amplifying PE3-targeted *FecB^B^* fragment in HEK293T cells.

| **ID** | **Sequence (5'-3')** | **Amplicon (bp)** |
| --- | --- | --- |
| *FecB^B^*_F | TTTGGATGGGAAAGTGGCGT | 226 |
| *FecB^B^*_R | TCGCAACGGGTCTAGACAGA |  |

**Table S2.** Sequences of pegRNA and sgRNA used in human HEK293T cells.

| ***FecB^B^*** | **Spacer sequence** | **3' Extension sequence** |
| --- | --- | --- |
| pegRNA | TCACCACAGAGGAAGCCAGC | GTCcGATATATTTCTGTCTCTCTGAACCAGCTGGCTTCCTCTGTG |
| sgRNA | TTCTGTCTCTGAACCAGC |  |

**Table S3.** Sequences of pegRNAs and sgRNAs used in sheep.

| **pegRNA** | **Spacer sequence** | **3' Extension sequence** |
| --- | --- | --- |
| *FecB^B^* | TCACTACAGAGGAGGCCAGC | GTCcGATATATTTCTGTCTCTCGGAACCAGCTGGCCTCCTCTGTA |
| *TBXT* | CACCGCTGGAAGTACGTGAA | TGCCCCaCGGCACCCACTCCCCGTTCACGTACTTCCAGC |
| **sgRNA** | **Spacer sequence** |  |
| *FecB^B^* | TTCTGTCTCTCGGAACCAGC |  |
| *TBXT* | GTCCAGCAGGAAGGAGTACA  GGGTGGATGTAGACGCAGCT |  |

**Table S4.** List of primers used for *in vitro* transcription.

| **Description** | **Sequence (5'-3')^*^** |
| --- | --- |
| *FecB^B^*-pegRNA | F:GATCCCTAATACGACTCACTATAGGTCACTACAGAGGAGGCCAGC |
|  | R: AAAAAAAATACAGAGGAGGCCAGCTGGT |
| *FecB^B^*-sgRNA | F:ATCCCTAATACGACTCACTATAGGTTCTGTCTCTCGGAACCAGC |
|  | R: AAAAAAAGCACCGACTCGGTGCCACTTTTTC |
| *TBXT*-pegRNA | F:GATCCCTAATACGACTCACTATAGGCACCGCTGGAAGTACGTGAA |
|  | R: AAAAAAAAGCTGGAAGTACGTGAACGGG |
| *TBXT*-sgRNA-1 | F:ATCCCTAATACGACTCACTATAGGGTCCAGCAGGAAGGAGTACA  R: AAAAAAAGCACCGACTCGGTGCCACTTTTTC |
| *TBXT*-sgRNA-2 | F:ATCCCTAATACGACTCACTATAGGGGGTGGATGTAGACGCAGCT |
|  | R: AAAAAAAGCACCGACTCGGTGCCACTTTTTC |

^*^Spacer sequences are underscored.

**Table S5.** List of primers for genotyping and amplifying PE3-targeted *BMPR1B* and *TBXT* fragments in newborn lambs.

| **Name** | **Sequence (5'-3')** | **Amplicon (bp)** |
| --- | --- | --- |
| *BMPR1B*_F | AGGTCCAGAGGACGATAGCA | 234 |
| *BMPR1B*_R | AGGAAACCCTGAACATCGCTAA |  |
| *TBXT*_F | GGCCCCTTTTAAGTTCCCAG | 440 |
| *TBXT*_R | ACAAGAAGGTGCAGAGTCACAG |  |

**Table S6.** List of predicted off-target sites for PE3-targeted *BMPR1B*.

| Position | 20 | 19 | 18 | 17 | 16 | 15 | 14 | 13 | 12 | 11 | 10 | 9 | 8 | 7 | 6 | 5 | 4 | 3 | 2 | 1 | N | G | G | Location | | | |
| --- | --- | --- | --- | --- | --- | --- | --- | --- | --- | --- | --- | --- | --- | --- | --- | --- | --- | --- | --- | --- | --- | --- | --- | --- | --- | --- | --- |
| spacer | T | C | A | C | T | A | C | A | G | A | G | G | A | G | G | C | C | A | G | C | T | G | G | Chr. | Start | End | Strand |
| OT1 | C | C | A | C | A | A | C | A | G | T | G | G | A | G | G | C | C | A | G | C | A | G | G | 5 | 102272469 | 102272491 | + |
| OT2 | C | C | A | C | T | A | C | A | G | T | T | G | A | G | G | C | C | A | G | C | A | G | G | 10 | 12288188 | 12288210 | + |
| OT3 | T | G | A | C | T | C | C | A | T | A | G | G | A | G | G | C | C | A | G | C | A | G | G | 12 | 46334548 | 46334570 | + |
| OT4 | T | C | C | C | A | A | C | A | G | A | G | G | A | G | T | C | C | A | G | C | T | G | G | 13 | 66084070 | 66084092 | + |
| OT5 | T | C | A | C | T | A | G | A | G | G | G | G | A | G | G | C | C | G | G | C | A | G | G | 24 | 37166519 | 37166541 | – |

| Position | 20 | 19 | 18 | 17 | 16 | 15 | 14 | 13 | 12 | 11 | 10 | 9 | 8 | 7 | 6 | 5 | 4 | 3 | 2 | 1 | N | G | G | Location | | | |
| --- | --- | --- | --- | --- | --- | --- | --- | --- | --- | --- | --- | --- | --- | --- | --- | --- | --- | --- | --- | --- | --- | --- | --- | --- | --- | --- | --- |
| nick | T | T | C | T | G | T | C | T | C | T | C | G | G | A | A | C | C | A | G | C | T | G | G | Chr. | Start | End | Strand |
| OT1 | T | T | C | T | G | T | C | T | T | T | G | G | G | A | A | C | C | A | G | C | T | G | G | 4 | 47418822 | 47418844 | + |
| OT2 | T | T | G | T | G | T | C | T | C | T | C | T | G | G | A | C | C | A | G | C | A | G | G | 5 | 6751810 | 6751832 | – |
| OT3 | T | A | C | T | G | T | C | T | C | C | A | G | G | A | A | C | C | A | G | C | T | G | G | 9 | 42069398 | 42069420 | – |
| OT4 | T | T | C | T | T | T | C | T | C | T | C | T | G | A | G | C | C | A | G | C | A | G | G | 11 | 1914241 | 1914263 | – |
| OT5 | T | T | C | T | C | T | C | T | C | T | C | G | G | A | A | T | C | A | G | C | A | G | G | 12 | 21173856 | 21173878 | + |
| OT6 | T | T | C | T | G | T | C | T | C | T | G | G | G | T | T | C | C | A | G | C | A | G | G | 12 | 74610462 | 74610484 | – |
| OT7 | T | T | C | A | G | T | C | T | C | T | C | T | C | A | A | C | C | A | G | C | T | G | G | 26 | 51683000 | 51683022 | – |
| OT8 | T | T | C | T | G | T | C | T | C | T | C | T | G | A | G | C | C | A | C | C | T | G | G | 26 | 59471499 | 59471521 | – |
| OT9 | G | T | C | T | G | T | C | T | C | T | C | T | G | A | A | C | C | A | G | C | T | G | G | 29 | 35173836 | 35173848 | – |

**Table S7.** List of predicted off-target sites for PE3-targeted *TBXT*.

| Position | 20 | 19 | 18 | 17 | 16 | 15 | 14 | 13 | 12 | 11 | 10 | 9 | 8 | 7 | 6 | 5 | 4 | 3 | 2 | 1 | N | G | G | Location | | | |
| --- | --- | --- | --- | --- | --- | --- | --- | --- | --- | --- | --- | --- | --- | --- | --- | --- | --- | --- | --- | --- | --- | --- | --- | --- | --- | --- | --- |
| spacer | C | A | C | C | G | C | T | G | G | A | A | G | T | A | C | G | T | G | A | A | C | G | G | Chr. | Start | End | Strand |
| OT1 | C | A | C | C | T | C | T | G | G | A | A | G | T | A | C | G | T | G | A | G | A | G | G | 29 | 7715686 | 7715708 | – |

| Position | 20 | 19 | 18 | 17 | 16 | 15 | 14 | 13 | 12 | 11 | 10 | 9 | 8 | 7 | 6 | 5 | 4 | 3 | 2 | 1 | N | G | G | Location | | | |
| --- | --- | --- | --- | --- | --- | --- | --- | --- | --- | --- | --- | --- | --- | --- | --- | --- | --- | --- | --- | --- | --- | --- | --- | --- | --- | --- | --- |
| nick1 | G | T | C | C | A | G | C | A | G | G | A | A | G | G | A | G | T | A | C | A | T | G | G | Chr. | Start | End | Strand |
| OT1 | G | G | C | C | A | G | C | A | G | G | C | A | G | G | T | G | T | A | C | A | G | G | G | 4 | 193028 | 193050 | – |
| OT2 | G | T | C | C | A | G | A | A | G | G | A | G | T | G | A | G | T | A | C | A | T | G | G | 4 | 120659716 | 120659738 | – |
| OT3 | G | T | C | C | T | G | C | A | G | G | A | A | G | A | A | G | G | A | C | A | G | G | G | 6 | 225851832 | 225851854 | – |
| OT4 | G | T | A | C | A | G | C | A | G | A | A | A | G | G | A | A | T | A | C | A | T | G | G | 8 | 90401952 | 90401974 | + |
| OT5 | G | A | C | C | A | G | C | A | G | G | A | A | G | G | A | G | A | A | G | A | G | G | G | 10 | 50239781 | 50239803 | – |
| OT6 | G | A | C | C | G | G | C | A | G | G | A | A | G | G | A | G | A | A | C | A | G | G | G | 13 | 78733110 | 78733132 | – |
| OT7 | G | G | C | C | A | G | A | A | G | G | A | A | A | G | A | G | T | A | C | A | G | G | G | 18 | 74062542 | 74062564 | + |
| OT8 | G | T | G | C | A | G | C | A | G | G | A | A | G | C | A | G | T | A | C | C | T | G | G | 20 | 12185024 | 12185046 | + |

| Position | 20 | 19 | 18 | 17 | 16 | 15 | 14 | 13 | 12 | 11 | 10 | 9 | 8 | 7 | 6 | 5 | 4 | 3 | 2 | 1 | N | G | G | Location | | | |
| --- | --- | --- | --- | --- | --- | --- | --- | --- | --- | --- | --- | --- | --- | --- | --- | --- | --- | --- | --- | --- | --- | --- | --- | --- | --- | --- | --- |
| nick2 | G | G | G | T | G | G | A | T | G | T | A | G | A | C | G | C | A | G | C | T | G | G | G | Chr. | Start | End | Strand |
| OT1 | G | G | G | T | G | G | A | A | G | T | A | G | A | C | C | C | A | G | A | T | G | G | G | 4 | 193987736 | 193987758 | + |
| OT2 | G | G | G | T | G | G | A | T | G | C | A | G | T | T | G | C | A | G | C | T | T | G | G | 4 | 263847105 | 263847127 | + |
| OT3 | G | G | G | T | A | C | A | T | G | T | A | G | A | C | A | C | A | G | C | T | A | G | G | 6 | 113900540 | 113900562 | + |
| OT4 | G | G | G | T | G | G | A | G | G | G | A | G | A | G | G | C | A | G | C | T | A | G | G | 15 | 1224029 | 1224051 | + |
| OT5 | C | G | G | T | G | G | A | T | G | C | A | G | A | G | G | C | A | G | C | T | G | G | G | 17 | 11399294 | 11399316 | – |
| OT6 | G | G | G | T | G | G | A | T | G | T | T | G | A | C | G | G | A | G | C | T | T | G | G | 17 | 22620635 | 22620657 | + |
| OT7 | G | G | G | T | G | G | A | T | G | T | A | G | A | C | T | C | A | G | C | T | G | G | G | 17 | 44159973 | 44159995 | – |

**Table S8.** List of primers for genotyping and amplifying predicted off-target site fragments in *FecB^B^*-edited sheep.

| **Name** | **Sequence (5'-3')** | **Amplicon (bp)** |
| --- | --- | --- |
| *BMPR1B*_OT1F | ACCCAGAGATGGAACCTGTG | 218 |
| *BMPR1B*_OT1R | ATCCGTCTCCAGACAGTTCT |  |
| *BMPR1B*_OT2F | CATTGTGTTCAAGGGGCCAG | 433 |
| *BMPR1B*_OT2R | AGGTGCAAAACTCCATCGCT |  |
| *BMPR1B*_OT3F | CTGTGTTCCCAGACCTCCTT | 292 |
| *BMPR1B*_OT3R | AAGACCCTGATGCTGGGATTG |  |
| *BMPR1B*_OT4F | GGAAGTTTGGTGAAAACGGGG | 475 |
| *BMPR1B*_OT4R | GCTCAGCTGGCAAAGAATCC |  |
| *BMPR1B*_OT5F | TTCAGACCCCTCATCAGCAAG | 191 |
| *BMPR1B*_OT5R | CAGACTACAGACCATGCGGT |  |
| *BMPR1B*_nick_OT1F | ATCCCAAGCCCCAAGTCTAA | 360 |
| *BMPR1B*_nick_OT1R | TCCAGGGGATCTTCCATACCC |  |
| *BMPR1B*_nick_OT2F | TGCCCTGCACCTCTGTAACT | 453 |
| *BMPR1B*_nick_OT2R | GAGCTTCAAATTCTGCCCCTG |  |
| *BMPR1B*_nick_OT3F | ATATCACCCAAGGGGAGCCA | 227 |
| *BMPR1B*_nick_OT3R | TCCTGAATGAGAACCCACAGA |  |
| *BMPR1B*_nick_OT4F | AAAGCACAGACTTCACTGCC | 461 |
| *BMPR1B*_nick_OT4R | TGTGTGACTCTTTGCGACCA |  |
| *BMPR1B*_nick_OT5F | AAAAGGCCCTGTGCTTCAGA | 483 |
| *BMPR1B*_nick_OT5R | CGTCCTCAACTCTTGCCGTA |  |
| *BMPR1B*_nick_OT6F | TGCCTTTAAAATATGGGGCTACG | 547 |
| *BMPR1B*_nick_OT6R | CCTGTATCCTGCCACCCAGA |  |
| *BMPR1B*_nick_OT7F | TATGCCACTGCCCAGTCTTTC | 379 |
| *BMPR1B*_nick_OT7R | TGCTTCTCAACCACCCTAGAC |  |
| *BMPR1B*_nick_OT8F | GTCGGATCAAGTGGAGGGAC | 306 |
| *BMPR1B*_nick_OT8R | TGAGACCTCCATGGGCTGTA |  |
| *BMPR1B*_nick_OT9F | CAACTCTGCGGTCACTTCCA | 229 |
| *BMPR1B*_nick_OT9R | GCACCTGGGGGAGCATTTAT |  |

**Table S9.** List of primers for genotyping and amplifying predicted off-target site fragments in *TBXT*-edited sheep.

| **Name** | **Sequence (5'-3')** | **Amplicon (bp)** |
| --- | --- | --- |
| *TBXT*_OT1F | GTGCAATTGAGCACTTCCTG | 605 |
| *TBXT*_OT1R | TGATGGGGTGCATCACAGTTTA |  |
| *TBXT*_nick1_OT1F | GGCCCCTTGTGTTTACCTCA | 465 |
| *TBXT*_nick1_OT1R | TGACTGAGCGACTGAACACC |  |
| *TBXT*_nick1_OT2F | CCCTGTGCATTTTCTGCACC | 339 |
| *TBXT*_nick1_OT2R | ATCCAATGGGCACCGAAGTT |  |
| *TBXT*_nick1_OT3F | AACTTATGCCCCCGGAACTG | 541 |
| *TBXT*_nick1_OT3R | GAATGAAGTCGCCCAAGGGA |  |
| *TBXT*_nick1_OT4F | TGAGCTAAATGTTCTCCCCGC | 440 |
| *TBXT*_nick1_OT4R | CGGACCTGTTTTCCACGGTT |  |
| *TBXT*_nick1_OT5F | AATTTGGACAGCTCCCAAGC | 430 |
| *TBXT*_nick1_OT5R | CCCCCATTCCCTTCCAACTAC |  |
| *TBXT*_nick1_OT6F | CCTTTCGTGGAGGGTACAAGG | 471 |
| *TBXT*_nick1_OT6R | AGACGCCAGTGAACACAACC |  |
| *TBXT*_nick1_OT7F | CCCTCAGCGACTCCACTTTC | 471 |
| *TBXT*_nick1_OT7R | CTGCTTGGAACTTTTCGGAGG |  |
| *TBXT*_nick1_OT8F | AACTTATGCCCCCGGAACTG | 414 |
| *TBXT*_nick1_OT8R | GAATGAAGTCGCCCAAGGGA |  |
| *TBXT*_nick2_OT1F | CTCGATGGAACATCTGGGGC | 345 |
| *TBXT*_nick2_OT1R | CTCAGCGAAGCAAATTGCCA |  |
| *TBXT*_nick2_OT2F | GTGTCTGTCATTTGGCGCAG | 365 |
| *TBXT*_nick2_OT2R | CAGAAGAGGATAAGGCGGGC |  |
| *TBXT*_nick2_OT3F | TAGGCCTGTTAAACACAGGAG | 377 |
| *TBXT*_nick2_OT3R | TCACACTGTCAACCTGTCCTC |  |
| *TBXT*_nick2_OT4F | GTCGGGAGACCTGGGATTTC | 601 |
| *TBXT*_nick2_OT4R | AAGTCCCTACGCAGTCTCCT |  |
| *TBXT*_nick2_OT5F | CTCAGAAAACCGCTGCCTGT | 504 |
| *TBXT*_nick2_OT5R | CTGGTGGGTAATCCGTGTCAT |  |
| *TBXT*_nick2_OT6F | AGAGACAGCCCTCTTGGTCT | 476 |
| *TBXT*_nick2_OT6R | GCGGTGGAAGGAAGATTATGC |  |
| *TBXT*_nick2_OT7F | CTCACCAACGAGATGACGGT | 336 |
| *TBXT*_nick2_OT7R | GTGCTCACTGCCATGAACTG |  |

**Table S10.** Sequences of pegRNAs and sgRNAs used in pigs.

| **pegRNA** | **Spacer sequence** | **3' Extension sequence** |
| --- | --- | --- |
| *KCNJ5* | CACCCGAAAGCCGTACCCGA | GACTGAGACGACCATCAGGTACGGCTTT |
| **sgRNA** | **Spacer sequence** |  |
| *KCNJ5* | ACTGAGACGACCATCAGGTA |  |

**Table S11.** List of primers for genotyping and amplifying PE3-targeted *KCNJ5* in porcine kidney fibroblasts and blastocysts.

| **Name** | **Sequence (5'-3')** | **Amplicon (bp)** |
| --- | --- | --- |
| *KCNJ5*-FW | TGGCTCTTCTTTGGCTTC | 344 bp |
| *KCNJ5*-RV2 | AGCTTCTCGTCCCGCA |  |
| *KCNJ5*-FW2 | GCCCGCGATTATATCCCCAT | 560 bp |
| *KCNJ5*-RV2 | AGCTTCTCGTCCCGCA |  |
| *KCNJ5*-FW5 | GCCCATGTCCCCTGTACACTA | 971 bp |
| *KCNJ5*-RV4 | TCTCGTGCGAGATGATGAGTG |  |

**Table S12.** Number of blastocysts generated *per* SCNT session using *KCNJ5^G151R/+^* porcine kidney fibroblasts as donor cells.

| SCNT Session | Complexes generated | Complexes cultured after activation | Number of blastocysts | Blastocyst rate |
| --- | --- | --- | --- | --- |
| 1 | 60 | 32 | 5 | 16% |
| 2 | 40 | 30 | 3 | 10% |
| 3 | 73 | 36 | 2 | 6% |
| Total | 173 | 98 | 10 | 10% |
